# Echogenic swirling pattern, carcinoembryonic antigen, and lactate dehydrogenase in the diagnosis of malignant pleural effusion

**DOI:** 10.1038/s41598-022-08188-y

**Published:** 2022-03-08

**Authors:** Chih-Feng Chian, Fu-Ping Wu, Chen-Liang Tsai, Chung-Kan Peng, Chih-Hao Shen, Wann-Cherng Perng, Shih-Chang Hsu

**Affiliations:** 1 Division of Pulmonary and Critical Medicine, Department of Internal Medicine, Tri-Service General Hospital, National Defense Medical Center, Taipei, Taiwan, ROC; 2 Hsiao Chung-Cheng Hospital, New Taipei City, Taiwan, ROC; 3 Hyperbaric Oxygen Therapy Center, Division of Pulmonary and Critical Care Medicine, Department of Internal Medicine, Tri-Service General Hospital, National Defense Medical Center, Taipei, Taiwan, ROC; 4 Graduate Institute of Medical Sciences, National Defense Medical Center, Taipei, Taiwan, ROC; 5 Emergency Department, Department of Emergency and Critical Medicine, Wan Fang Hospital, Taipei Medical University, Taipei, Taiwan, ROC; 6 Department of Emergency, School of Medicine, College of Medicine, Taipei Medical University, No.250, Wuxing St., 11031 Taipei, Taiwan

**Keywords:** Cancer, Oncology, Diagnosis, Medical imaging

## Abstract

The echogenic swirling pattern has a role in predicting malignant pleural effusion (MPE). However, its predictive ability is suboptimal, and its clinical utility remains to be defined. The aim of this study was to assess the diagnostic potential of the echogenic swirling pattern combined with pleural carcinoembryonic antigen (CEA) and routine laboratory tests of pleural effusion in MPE. The 80 consecutive patients with underlying malignancy and pleural effusions were recruited. All patients underwent one diagnostic thoracentesis with a cytologic examination of pleural fluid. Our study showed that the sensitivity of echogenic swirling patterns in MPE diagnosis was 67.7%, specificity was 72.2%, positive predictive value (PPV) was 89.4%, and negative predictive value (NPV) was 39.4%. Both CEA and lactate dehydrogenase (LDH) had acceptable sensitivity (71.0% and 60.7%) and specificity (72.2% and 77.8%). Combining the echogenic swirling pattern, pleural CEA, and pleural LDH, the highest sensitivity (95.2%) with a good PPV (86.8) was reached. In this clinical study, we found that combining the echogenic swirling pattern, pleural CEA, and pleural LDH had a higher sensitivity and a high positive predictive value for the diagnosis of MPE. This combination is a potentially suitable method for MPE screening in cancer patients with pleural effusions.

## Introduction

For patients with underlying malignancy, a diagnosis of pleural effusion affects the standard of care and is challenging for physicians. In such cases, the causes of pleural effusion include tumor cells that have invaded the pleura, hypoalbuminemia, mediastinal lymph node tumor infiltration, and postobstructive pneumonia^1,2^. The presence of MPE can affect the tumor stage and prediction of prognosis. For example, patients with non-small-cell lung cancer and MPE are classified as stage IV, which indicates an unresectable state and a short median survival time. In clinical practice, the first step in diagnosing MPE is thoracentesis with a cytologic examination of pleural fluid. However, the diagnostic rate is only 60% on average in general categories of patients with MPE^3,4^. For patients without positive malignant cells in the pleural fluid, image-guided pleural biopsy or thoracoscopic biopsy of the pleura are recommended procedures for identifying MPE^2,5,6^. Although these methods can improve the diagnostic sensitivity, they are invasive with the risks of complications and are technically demanding with a high cost. Hence, establishing a practical and economical method for the diagnosis of MPE has significant clinical value.

Real-time chest ultrasonography offers a more accurate detection of a minimal volume of fluid than traditional radiography^7^. Ultrasound-guided diagnostic thoracentesis is also a simple, safe, and well-tolerated method in patients with severe illness or on mechanical ventilation^8–10^. Some sonographic findings suggest that MPE includes pleural nodules, hypoechoic pleural thickening with irregular or unclear borders, or a swirling pattern within the pleural fluid^11–13^. Under real-time chest sonography, the echogenic swirling pattern is defined as floating echogenic particles within the pleural effusion that have circular movement in response to respiratory movement or heartbeat. Our previous study found that the positive predictive rate of echogenic swirling patterns in MPE was 81.8%. Our finding was also highlighted in a well-known textbook^14^.

However, the sensitivity and specificity of echogenic swirling patterns in MPE could not be determined in that retrospective study^12^. The presence of tumor markers, such as CEA, carbohydrate antigen 15-3, cytokeratin 19 fragments, and cancer antigen 125, in the pleural fluid has been suggested as a complementary method to select patients for further invasive procedures to establish the diagnosis of MPE^15–18^. However, the routine determination of a panel of tumor markers in all patients with malignancy and pleural fluid cannot be done in every hospital. Measurement of the CEA value in the pleural fluid has been one of the most predictive single markers in the differentiation of malignant pleural effusion from nonmalignant pleural effusion^19^. The measurement of a single marker is more convenient than the measurement of a panel of tumor markers in the diagnosis of MPE. Additionally, the routine analytical parameters of pleural effusion, such as the percentage of neutrophils, the percentage of lymphocytes, total proteins and lactate dehydrogenase (LDH), can provide useful information in distinguishing between MPE and parapneumonic pleural effusion (PPE)^20,21^.

Thus, in the present study, we tried to combine the echogenic swirling pattern with other available standard parameters for the differential diagnosis of MPE. The objective of this study was to evaluate the predictive ability of the echogenic swirling pattern plus other standard parameters of pleural effusion in the diagnosis of MPE.

## Results

### Tumor origin of cancers

We consecutively recruited one hundred patients with pleural effusion from April 2007 to June 2008 at Tri-Service General Hospital, a tertiary referral center in Taiwan. Twenty subjects who initially had negative cytologic examinations without further diagnostic procedures were excluded from the analysis because eighteen of them had a short lifetime within one month and the other two died in 5 and 7 months and had persistent, large pleural effusions. Thus, eighty subjects were enrolled for the final data analyses. The distribution of the tumor origin of cancers is shown in Table 1.

**Table 1:** Distribution of the type of neoplasm in 80 patients.

Neoplastic types	No
**Gum**	
Squamous cell carcinoma	1
**Tongue**	
Squamous cell carcinoma	1
**Parotid gland**	
Carcinosarcoma	1
**Hypopharynx**	
Squamous cell carcinoma	2
**Thyroid**	
Follicular carcinoma	1
Papillary carcinoma	1
Nasopharyngeal carcinoma	2
**Lung**	
Adenocarcinoma	25
Small cell carcinoma	2
**Breast**	
Invasive ductal carcinoma	10
Mucinous cell carcinoma	1
Signet ring cell carcinoma	1
**Esophagus**	
Squamous cell carcinoma	1
**Stomach**	
Adenocarcinoma	6
**Pancreas**	
Adenocarcinoma	1
**Liver**	
Hepatocellular carcinoma	3
**Colon**	
Adenocarcinoma	5
**Rectum**	
Adenocarcinoma	3
**Cervix**	
Squamous cell carcinoma	1
Adenocarcinoma	1
**Ovary**	
Serous papillary cystadenocarcinoma	1
Mixed epithelial type cystadenocarcinoma	1
Adenocarcinoma	1
Lymphoma	3
Multiple myeloma	1
Unknown origin	4

MPE was diagnosed in 62 patients: by cytologic examination of pleural fluid in 50 patients, by pleural biopsy in 3 patients, by VATS in 4 patients, and by pleural nodules on computed tomography of the chest in 5 patients. Of these latter 5 patients, two patients exhibited the echogenic swirling pattern, and three patients had no detectable echogenic swirling pattern. Eighteen patients without a diagnosis of MPE were followed for at least 3 months after study enrollment and did not exhibit persistent pleural effusions. Of the eighteen patients with benign pleural effusion, five individuals had an unknown cause of pleural effusion. The etiologies of pleural effusion for the rest of the patients were as follows: pneumonia-related pleural effusion: 3; TB-related pleural effusion: 2; surgery-related pleural effusion: 2; congestive heart failure-related pleural effusion: 2; acute cholangitis-related pleural effusion: 2; cirrhosis-related pleural effusion: 1; and renal insufficiency-related pleural effusion: 1.

### Echogenic swirling pattern and malignant pleural effusions

The demographic, sonographic, and pleural fluid data of patients with MPE and non-MPE are shown in Table 2. A total of 80 individuals were included in the analysis, and the sex ratio significantly differed between MPE patients and non-MPE patients (p < 0.05). The presence of the echogenic swirling pattern in the MPE and non-MPE groups was seen in 42 (67.74%) and 5 (27.78%) individuals, respectively (p < 0.01). In terms of pleural fluid analysis, total protein (p < 0.05), glucose (p < 0.05), LDH (p < 0.01), and CEA (p < 0.01) levels were significantly higher in the MPE group than in the non-MPE group.

**Table 2:** Comparisons of demographic, sonographic and pleural fluid data between patient with MPE and those with non-MPE.

Variable	MPE (n = 62 )	Non-MPE (n = 18)	p value
**Demographic**			
Age, years (SD)	64.6 (14.6)	63.0 (16.9)	0.5644
Male (%)	26 (41.94%)	14 (77.78%)	0.0159
**Sonography**			
Swirling pattern (%)	42 (67.74%)	5 (27.78%)	** < 0.01**
**Pleural fluid**			
WBC, cells/µL (SD)	1691.7 (1762.0)	1598.6 (2147.9)	0.3076
Neutrophiles, % (SD)	14.46 (16.68)	23.12 (25.83)	0.432
Lymphocytes, % (SD)	47.26 (22.55)	45.83 (24.69)	0.8572
Protein, g/dL (SD)	4.53(0.97)	3.78 (1.13)	0.0432
Glucose, mg/dL (SD)	102.41 (45.47)	132.67 (63.45)	0.0397
LDH, U/L (SD)	525.4 (661.6)	293.7 (406.3)	** < 0.01**
CEA, ng/mL (SD)	194.2 (516.5)	10.8 (18.0)	** < 0.01**

Significant values are in bold.

**Figure 1:**
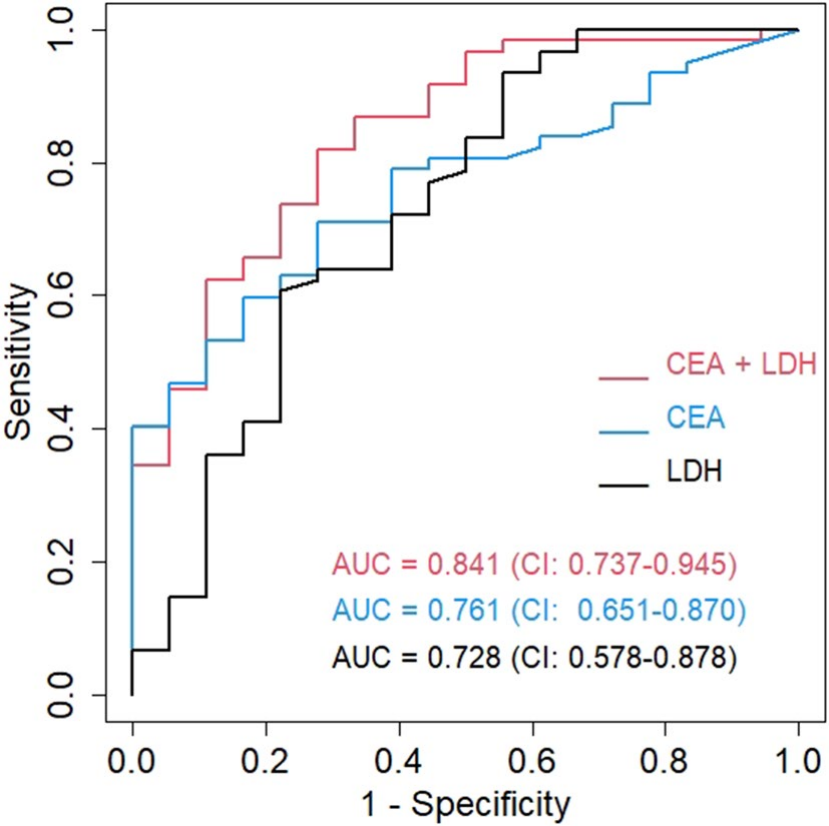
Receiver operating characteristic (ROC) curves for the prediction of MPE.

The CEA levels in pleural fluids ranged from 1.0 to 1985.0 ng/ml. The area under the curve of the receiver operating characteristic (ROC) curve was 0.761 (95% CI 0.651–0.870) (Fig. 1). A cutoff value of 4.455 ng/ml yielded a sensitivity of 71.0% and a specificity of 72.2%. The LDH concentration in pleural fluids ranged from 71 to 13,878 U/L. The area under the ROC curve was 0.728 (95% CI 0.587–0.878) (Fig. 1). A cutoff value of 250.5 U/L yielded a sensitivity of 60.7% and a specificity of 77.8%. By combining CEA and LDH levels, the area under the ROC curve increased to 0.841 (95% CI 0.737–0.945) (Fig. 1). We determined that the sensitivity of the swirling pattern in the diagnosis of MPE was 67.7%. Its specificity, positive predictive value, and negative predictive value were 72.2%, 89.4%, and 39.4%, respectively, as calculated from its frequency in MPE and non-MPE participants (Table 3).

**Table 3:** Comparing different methods for predicting patient diagnosis status.

	Sensitivity (%)	Specificity (%)	PPV (%)	NPV (%)
Swirling pattern (+)	67.7	72.2	89.4	39.4
CEA > 4.455	71.0	72.2	89.8	41.9
LDH > 250.5	60.7	77.8	90.2	36.8
Swirling pattern (+) or CEA > 4.455	88.7	55.6	87.3	58.8
Swirling pattern (+) or LDH > 250.5	82.3	61.1	87.9	50.0
CEA > 4.455 or LDH > 250.5	90.3	66.7	90.3	66.7
Swirling pattern (+) or CEA > 4.455 or LDH > 250.5	95.2	50.0	86.8	75.0

*PPV* positive predictive value, *NPV* negative predictive value.

### Combining different parameters in the diagnosis of MPE

In this study, we compared different combinations of predictive parameters for MPE diagnosis. The sensitivity, specificity, PPV, and NPV were calculated for the comparison. The results are presented in Table 3. Overall, when combining the different parameters, the sensitivity increased, and the specificity decreased. By combining all three parameters (echogenic swirling pattern, CEA, and LDH), the highest sensitivity (95.2%) was achieved.

## Discussion

In this prospective study, we determined that the sensitivity, specificity, PPV, and NPV of the echogenic swirling pattern in patients with MPE were 67.7%, 72.2%, 89.4%, and 39.4%, respectively. We also evaluated the predictive potential of LDH and CEA in pleural fluid in the diagnosis of MPE. Similar to the echogenic swirling pattern, both LDH and CEA had acceptable sensitivity and specificity. By combining the echogenic swirling pattern, pleural CEA, and pleural LDH, the highest sensitivity (95.2%) with a good PPV (86.8%) was reached.

In our previous retrospective study, for patients with pleural fluid and underlying malignancy, the diagnostic rate of MPE for patients presenting the echogenic swirling pattern was 81.8%. However, in that retrospective study, the sensitivity and specificity could not be determined because the malignant status of pleural effusions was unknown. In this study, we found that the sensitivity of the echogenic swirling pattern in MPE was only 67.7%, so it was an insufficient screening tool for patients with MPE. The specificity of the echogenic swirling pattern was 72.2%. In cancer patients without the echogenic swirling pattern in pleural fluid, the possibility of MPE could not be confidently excluded. However, the positive predictive value of the echogenic swirling pattern in MPE was 89.4%. Our study showed that seven patients with initial negative cytologic examinations were later diagnosed with MPE by pleural biopsy or VATS. Five of them presented the echogenic swirling pattern, and the other two showed high pleural CEA (23.44 ng/ml and 175.86 ng/ml). Moreover, our ROC curve analysis suggested that combining CEA and LDH in pleural fluids had the highest AUC in MPE prediction. Interestingly, recent studies also demonstrate that CEA and LDH in pleural fluid are potential biomarkers for the diagnosis of MPE^19,22,23^.

The cytology of pleural effusion and pleural biopsy are still the gold standards for diagnosing MPE^24^. Although both methods have excellent specificity, low sensitivity is a major drawback. As a result, the patient may need to undergo a VATS examination, multiple thoracenteses, or multiple pleural biopsies. However, currently, there are no definite guidelines for deciding which patients should undergo invasive diagnostic procedures. Since our method is a predictive procedure, it can potentially be used as a screening tool. The presence of an echogenic swirling pattern and elevated CEA or LDH in the pleural fluid of a cancer patient may suggest a high probability of MPE and indicate that additional tests are warranted despite initial negative cytologic examinations.

The measurement of a panel of tumor markers may have some clinical value in the diagnosis of MPE. Some patients with abnormal tumor markers in the pleural fluid would benefit from invasive procedures, such as thoracoscopy. However, routine use of a panel in the diagnosis of MPE has not been recommended because the clinical characteristics of patients were at least as predictive as the panel of tumor markers^25,26^. In this study, we measured pleural CEA, one of the common tumor markers in MPE diagnosis, instead of a panel^19^. The sensitivity of pleural CEA in MPE diagnosis was in an acceptable range. Our study suggests that a high pleural CEA value may indicate a diagnosis of MPE. However, high CEA levels have also been found in 9% of nonmalignant pleural effusions, especially in patients with parapneumonic effusions or empyema^27^.

Additional diagnostic tools to traditional cytologic examination for improving MPE diagnosis include imaging, image-guided lung biopsy, measurement of a panel of tumor markers, repeated thoracentesis, and detection of DNA methylation or aneuploidy in pleural fluid^2,10,28^. Most of these methods require specific laboratory analysis or high-end equipment, limiting their clinical applications. Real-time ultrasonography of the chest is a convenient method to detect pleural effusion, and its techniques have been intensively reviewed^11,29–31^. In this study, we showed the possibility of combining the echogenic swirling pattern, pleural CEA, and pleural LDH in MPE diagnosis. The issue of combining more than two different tests in MPE diagnosis is still uncertain. There may be a potential role of using a screening test for MPE.

Although this is a consecutive clinical study, there were still some limitations in this study. First, the sample size of this study was relatively small. However, the positive predictive value of MPE in cancer patients with the echogenic swirling pattern was similar to our previous results (89.4% and 81.8%, respectively)^12^. Second, twenty (20%) of the recruited patients were not included in the final data analysis because 18 subjects lived less than 30 days and died in the same hospitalization. Although the dropout percentage from this study was high, it may reflect real clinical practice, as some patients came to the hospital in their terminal stage. These patients could not tolerate very invasive procedures for the diagnosis of MPE and had a short lifespan. The other two subjects survived 5 and 7 months after recruitment. Both patients died in the hospital with a persistent, large volume of pleural effusion. Since they did not undergo further examinations for MPE, we could not determine their malignant status.

In conclusion, the positive predictive value of the echogenic swirling pattern in the diagnosis of MPE was 89.4%, and the echogenic swirling pattern correlated strongly with MPE in patients with malignancies. Pleural CEA and pleural LDH also have a reasonable specificity and positive predictive value in the diagnosis of MPE for cancer patients with pleural effusions. In this clinical study, we found that combining the echogenic swirling pattern, pleural CEA, and pleural LDH had a higher sensitivity and a higher positive predictive value for the diagnosis of MPE. Thus, this combination is a potentially good method for screening MPE in cancer patients with pleural effusions.

## Methods

### Patients and study design

We conducted a prospective study to determine the clinical application of the echogenic swirling pattern, CEA, and analytical parameters of pleural effusion for patients with underlying malignancy. The inclusion criterion was patients with pleural fluids and underlying malignancy. At first, two investigators recorded the sonographic findings at the same time to see whether the echogenic swirling pattern was present. Then, the patients underwent one diagnostic thoracentesis, and the pleural fluids were subjected to cytologic examinations, routine laboratory tests and CEA measurements. If cytologic examinations could not diagnose MPE, the next step for the diagnosis of MPE was decided by the patient's attending physician. If the patient's attending physician arranged pleural biopsies or video-assisted thoracoscopic surgical (VATS) examination, the diagnosis of MPE depended on the pathologic confirmation of the pleura. If patients did not undergo pleural biopsy or VATS as their final diagnostic procedures, however, they underwent another diagnostic thoracentesis in one month, and positive malignant cells in the pleural fluid were recognized as MPE. To define the nature of the pleural fluid, patients who underwent diagnostic thoracentesis with negative malignant cells in the pleural fluid and without further VATs or pleural biopsy were followed for at least the next three months. The patient was categorized as having paramalignant pleural effusions when he or she exhibited negative cytologic examination results and remained alive for at least 3 months without the existence of pleural effusion. Permission was received from all patients before diagnostic thoracentesis. The study was approved by the Institutional Review Board of Tri-Service General Hospital (TSGH-096-05-0016), and informed consent was obtained from the patients or their surrogates. All methods were carried out in accordance with relevant guidelines and regulations.

Malignant pleural effusions were defined as evidence of malignant cells on cytologic examination of pleural fluids or pathologic examination of pleural tissues. Paramalignant pleural effusions were defined as effusions that were not a direct result of neoplastic involvement of the pleura but were still related to the primary tumor. In this study, patients with negative cytologic examinations but presenting pleural nodules on computed tomography were classified as having MPE.

### Ultrasonographic criteria for defining the echogenic swirling pattern

Examinations were performed using a real-time ultrasound scanner (Toshiba SSA-340A; Toshiba; Tokyo, Japan) with 3.75 MHz sector transducers. All patients were examined in an upright position or the lateral decubitus position. Two well-trained chest physicians determined whether the echogenic swirling pattern was present during chest sonography. The echogenic swirling pattern was defined as numerous floating echogenic particles within the pleural effusion that moved in response to respiratory movement or heartbeat under real-time sonographic examination.

### Tumor marker assay

CEA levels were measured using a commercial radio immunoradiometric assay kit (CIS ELSA2-CEA, CIS Bio International, France).

### Statistical analysis

Categorical data are expressed as frequencies (%) and were tested with the chi-square test. Continuous variables are expressed as the mean with standard deviation (SD) and were compared by the Mann–Whitney U test. Candidate variables with a p value < 0.01 after statistical analysis were included in the following analysis (swirling pattern: p = 0.058; LDH: p = 0.0034; CEA: p = 0.00079). In the receiver operating characteristic (ROC) curve analysis, the area under the curve (AUC) and cutoff values (determined by Youden indices) were calculated. To evaluate the predictive ability of variables for MPE diagnosis, the sensitivity, specificity, positive predictive value, and negative predictive value were also calculated. Statistical analysis of the data obtained in the study was performed using R 4.1.0 software (R Foundation for Statistical Computing, Vienna, Austria). All statistical tests were two-sided using a significance level of 0.05.
